# CRISPR/Cas9 application in cancer therapy: a pioneering genome editing tool

**DOI:** 10.1186/s11658-022-00336-6

**Published:** 2022-05-04

**Authors:** Sadegh Shojaei Baghini, Zhanna R. Gardanova, Saeme Azizi Hassan Abadi, Burhan Abdullah Zaman, Ahmet İlhan, Navid Shomali, Ali Adili, Roozbeh Moghaddar, Amirhossein Fakhre Yaseri

**Affiliations:** 1 Plant Biotechnology Department, National Institute of Genetic Engineering and Biotechnology (NIGEB), Tehran, Iran; 2 Department of Psychotherapy, Pirogov Russian National Research Medical University, 1 Ostrovityanova St., 117997 Moscow, Russia; 3 Department of Nursery and Midwifery, Faculty of Laboratory Science, Islamic Azad University of Chalous, Mazandaran, Iran; 4 Basic Sciences Department, College of Pharmacy, University of Duhok, Kurdistan Region, Iraq; 5 Department of Medical Biochemistry, Faculty of Medicine, Cukurova University, Adana, Turkey; 6 Immunology Research Center (IRC), Tabriz University of Medical Sciences, Tabriz, Iran; 7 Department of Oncology, Tabriz University of Medical Sciences, Tabriz, Iran; 8 Senior Adult Oncology Department, Moffitt Cancer Center, University of South Florida, Tampa, USA; 9 Department of Pediatric Hematology and Oncology, School of Medicine, Ahvaz Jundishapur University of Medical Sciences, Ahvaz, Iran; 10 Faculty of Medicine, Qazvin University of Medical Sciences, Qazvin, Iran

**Keywords:** Genome editing, Clustered regularly interspaced short palindromic repeats (CRISPR), CRISPR associated protein 9 (Cas9), Cancer treatment

## Abstract

The progress of genetic engineering in the 1970s brought about a paradigm shift in genome editing technology. The clustered regularly interspaced short palindromic repeats/CRISPR associated protein 9 (CRISPR/Cas9) system is a flexible means to target and modify particular DNA sequences in the genome. Several applications of CRISPR/Cas9 are presently being studied in cancer biology and oncology to provide vigorous site-specific gene editing to enhance its biological and clinical uses. CRISPR's flexibility and ease of use have enabled the prompt achievement of almost any preferred alteration with greater efficiency and lower cost than preceding modalities. Also, CRISPR/Cas9 technology has recently been applied to improve the safety and efficacy of chimeric antigen receptor (CAR)-T cell therapies and defeat tumor cell resistance to conventional treatments such as chemotherapy and radiotherapy. The current review summarizes the application of CRISPR/Cas9 in cancer therapy. We also discuss the present obstacles and contemplate future possibilities in this context.

## Introduction

Genome editing tools have offered great advantages to the biological sciences [1, 2]. Various techniques, including zinc finger endonuclease (ZFN), transcription activator-like effector nuclease (TALEN), and the clustered regularly interspaced short palindromic repeats/CRISPR associated nuclease (CRISPR/Cas) system, have now been developed to provide efficient gene editing to enable treatment for cancers as well as infectious and genetic disorders [3, 4]. Moreover, genome editing tools offer new opportunities in basic cancer research and diagnosis, including wide advantages such as simple design, rapid operation, low cost, and robust scalability, introducing CRISPR/Cas as a rapidly evolving editing approach that is applicable to almost all genomic targets [5–7]. Historically, the term “CRISPR” was proposed by Mojica and Ruud Jansen (2001) [8]; such palindromic repeats were first recognized in *Escherichia coli* by Ishino et al. (1987) [9]. The function of these sequences remained unclear until 2005. Mojica et al. (2005) first stated that CRISPR serves a significant role in the bacterial immune system [10]. Molecular reports have shown that CRISPR repeats could be detected in around 40% of bacteria and about 90% of archaea [11].

During the last two decades, oncogenes, tumor suppressor genes, metabolism-related genes, and genes involved in resistance to chemo- and radiotherapy have been targeted and edited by using the CRISPR/Cas9 system to constrain tumor growth and progression [12–15]. Moreover, CRISPR/Cas9-mediated genome editing has wide-ranging potential in cancer therapy. Tumorigenesis is a complicated process including complex interactions between cancer cells and the host immune system [16]. Integration of CRISPR/Cas technique with cancer immunotherapy, such as chimeric antigen receptor (CAR)-T cell-based therapy, and its ability to alleviate carcinogenic viral infections such as human papillomavirus (HPV) has recently emerged as a promising therapeutic approach to a wide range of diseases [17, 18]. Nonetheless, the off-target activity of the CRISPR/Cas9 genome editing tool has been a significant drawback [19]. Therefore, improving its specificity to overcome such off-target effects for safe therapeutic application of CRISPR/Cas9 is of great importance. This study emphasizes recent findings concerning the application of CRISPR/Cas9 (type II CRISPR/Cas9) methods in cancer therapy. We also discuss existing hurdles and contemplate future directions. Furthermore, a glimpse of the ability of the CRISPR/Cas9 system to evolve “off-the-shelf” CAR-T cells with higher anticancer competence is also presented.

## CRISPR/Cas9 systems

The growth of artificially designed meganucleases (homing endonucleases) followed by ZFNs and TALENs, and CRISPR/Cas9 has promoted the efficacy of gene editing tools, providing groundbreaking developments in site-specific nuclease (SSN) systems [3]. However, the main drawbacks of cloning and engineering of ZFNs and TALENs have limited their application by the scientific community [20]. In this light, CRISPR technology has renewed SSN systems, resulting in deep editing efficacy and simplicity even for minimal sequences and thus becoming a preferred tool for various genome-targeting goals [21, 22].

### Action mechanism

**Fig. 1:**
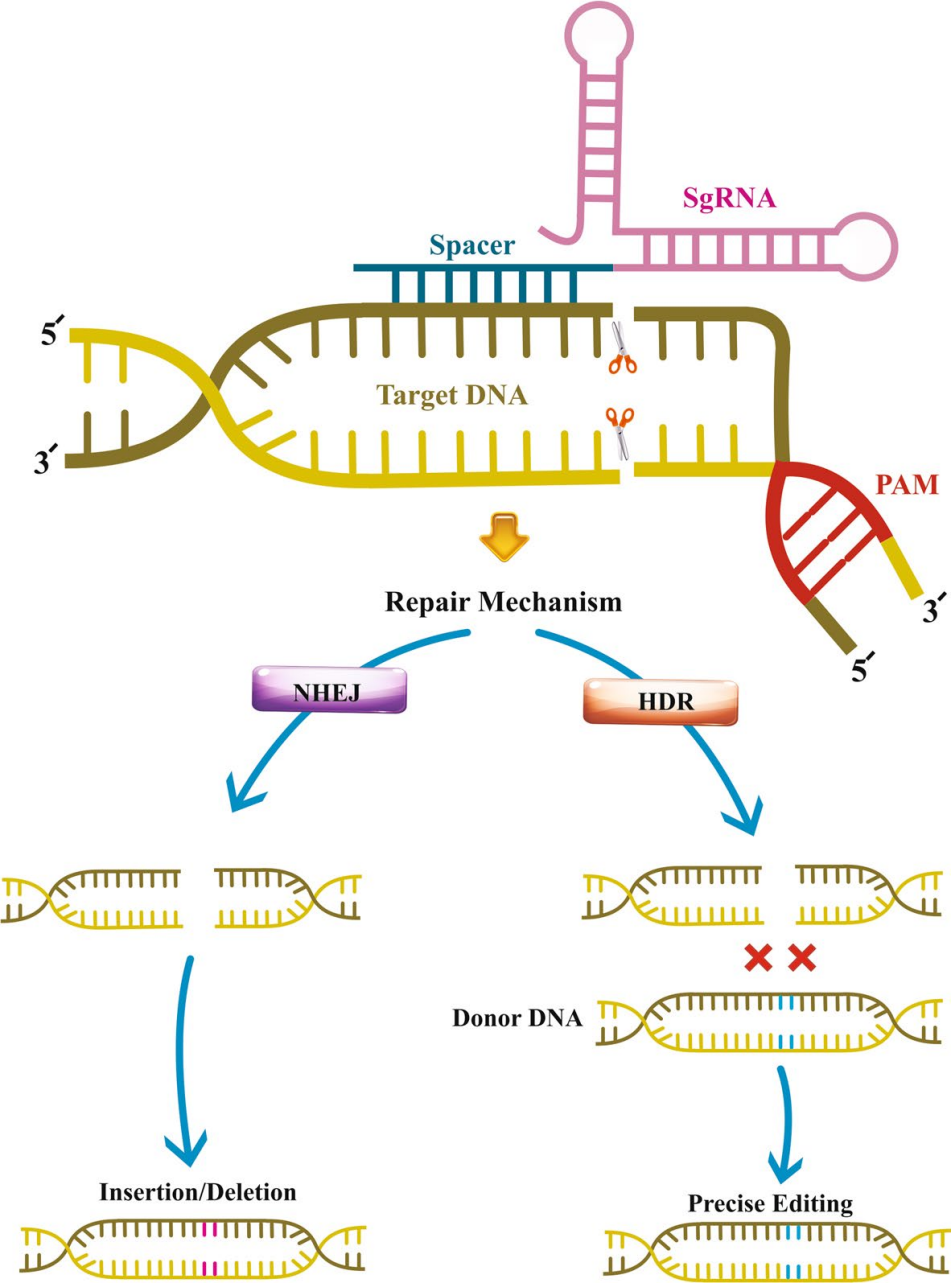
Action mechanism of CRISPR/Cas9 system, including nonhomologous end-joining (NHEJ), homology-directed repair (HDR), single-guide RNA (sgRNA), and protospacer adjacent motif (PAM)

It is now known that bacteria catch snippets of DNA from invading viruses and integrate them into their genome to generate CRISPR arrays, enabling bacteria to become familiar with viruses for their next possible encounter. In response to a subsequent invasion, the bacteria use RNA fragments from such CRISPR arrays to affect the DNA of the viruses [23]. The bacteria then exploit Cas9 or a similar enzyme (e.g., Cas3 and Cas10) to cut the DNA segment, thereby limiting the viability and dangerous functions of the virus. Mechanically, the natural CRISPR/Cas9 system in bacteria consists of two main RNA segments: mature CRISPR RNA (crRNA) and trans-activating crRNA (tracrRNA) [24, 25]. A functional guide RNA (gRNA) is produced by pairing the tracrRNA base with the crRNA. The crRNA sequence can be separated into guide and repeat regions, whereas the tracrRNA sequence includes an anti-repeat region and three stem-loop assemblies. The guide region yields the gRNA:DNA heteroduplex by Watson and Crick base pairing with the DNA target site. The repeat and anti-repeat regions establish the repeat:anti-repeat duplex by Watson and Crick base pairing [24, 26]. Notably, while Cas9 applies the tracrRNA part of the guide as a handle, the crRNA spacer segment directs the complex for identifying viral sequences [27]. Indeed, crRNA and tracrRNAs form Cas9 protein–RNA machinery that cuts the viral sequence with DNA double-strand breaks (DSBs). One of the advantages of this two-component system is that the gRNA can be altered independently from the Cas nuclease, facilitating the modification of CRISPR as a genome editing tool with unrestricted target capability and high efficiency [28, 29]. In contrast to conventional tandem repeats in the genome, CRISPR repeat clusters are separated by nonrepeating DNA sequences termed spacers belonging to dangerous viruses [10, 30]. There is substantial similarity between the spacer sequences and the protospacer-adjacent motif (PAM) sequences targeted by the guide RNA [31]. PAMs are short DNA sequences (typically 2–6 base pairs in length) situated 3–4 nucleotides downstream from the cleavage site. *Streptococcus pyogenes* Cas9 (SpCas9) nuclease is directed by a sgRNA to a 20-bp sequence of target DNA located next to a three-base-pair PAM (5′-NGG-3′), providing a blunt-ended DNA double-strand break (DSB). DSBs stimulate cellular repair systems, chiefly nonhomologous end-joining (NHEJ, imprecise repair) or homology-directed repair (HDR, precise repair) (Fig. 1). In this regard, CRISPR has become known as a powerful, reprogrammable genome editing tool. CRISPR technology includes an endonuclease such as Cas9 protein concomitant with a single sgRNA, which is functionally comparable to the crRNA-tracrRNA complex in bacteria. The sgRNA plays a paramount role in determining the specificity and cutting activities of the endonuclease [32–34].

### Classes and types

A series of Cas9 variants have been industrialized to improve the editing fidelity or targeting range of CRISPR/Cas9 (Table 1). Regarding the organization of the effector protein and the presence or lack of signature genes, conservation of the protein sequence, and organization of respective genomic loci, CRISPR systems can mainly be classified into 2 main classes, 6 types, and over 30 subtypes [8]. Class 1 consists of type I and type III CRISPR systems and is typically found in Archaea, while class 2 includes type II, IV, V, and VI CRISPR systems [35, 36]. Class 2 includes only one effector protein, while class 1 comprises multi-subunit Cas protein complexes. Importantly, a unified classification of these systems should be based on various criteria because of the complexity of the genomic architectures and the rapid evolution of CRISPR/Cas systems [36]. Significantly, three specific signature genes distinguish the three central CRISPR systems: Cas3 in type I systems, Cas9 in type II, and Cas10 in type III. Despite the introduction of several CRISPR/Cas systems for gene editing applications, the most broadly used type is the type II CRISPR-Cas9 system from *S. pyogenes*. In addition, Cpf1 protein derived from AsCpf1 (Acidaminococcus sp.) and LbCpf1 (Lachnospiraceae bacterium) has attracted increasing attention [37, 38]. In general, class 2 systems have more capacity to improve genome editing and genetic screening, as confirmed by several reports using the Cas9 (Csn1), Cas12a (Cpf1), Cas13a (C2c2), and Cas13b (C2c6) systems.

**Table 1:** Cas9 variants

Variant	PAM sequence (5′–3′)	Utilization	Ref.
SpCas9	NGG	Multiplex genome editing in mammalian cells	[265]
SpCas9-VRER	NGCG	Editing previously inaccessible sites in zebrafish embryos as well as human cells	[269]
SaCas9	NNGRRT	More efficient genome edition by the AAV-SaCas9-gRNA vector system	[270]
CjCas9	NNNVRYM	In vivo genome edition within muscles of dystrophin KO mice	[271]
SpCas9-NG	NG	More efficient and accurate genome edition in mouse zygotes and also somatic culture cells	[272]
evoCas9	NGG	Restricting unspecific cleavage of a difficult-to-discriminate off-target region and fully perturbing the cleavage of two additional off-targets	[273]
xCas9–3.7	NG, GAA, GAT	Base replacement of C.G → T.A and A.T → G.C for pathogenic mutation sites	[274]
SpRY	NA	Exact editing extending to almost the whole genome	[275]

*Pyogenes* Cas9 (SpCas9), Small Cas9 ortholog from *Staphylococcus aureus* (SaCas9), *Campylobacter jejuni* Cas9 (CjCas9), adeno-associated viral (AAV) vectors, protospacer adjacent motif (PAM), Not applicable (NA)

## CRISPR/Cas9 applications in viral infections and genetic disorders

### Viral infection

The CRISPR/Cas9 tool can be applied not only to modify particular nucleotide sequences in the human genome but also to target the double-stranded DNA (dsDNA) of viruses [39]. Interestingly, the CRISPR/Cas9 machinery can be equipped with multiple sgRNAs, which facilitates action on various genomic loci in a single cell by Cas9 endonucleases [40, 41]. Cas9 variants also enable targeted gene mutation, transcriptional activation and suppression, epigenetic alteration, imaging of DNA loci, and single-base mutations [28, 42]. By using the CRISPR/Cas9 system, clearance of viruses from infected cells becomes hypothetically practical for any DNA- or RNA-mediated virus during their pathological process. Therefore, the CRISPR/Cas9 technique has become a game-changing tool for modifying several developmental phases of the viral life cycle and holds the capacity to enable efficient genetic therapy versus human viruses (Table 2) [43, 44]. In recent years, CRISPR/Cas9-mediated antiviral protocols to manipulate infectious human viruses have been applied efficiently. In this regard, the CRISPR/Cas9 system has shown remarkable efficacy against human immunodeficiency virus (HIV), hepatitis B virus (HBV), and HPV [45, 46].

**Table 2:** CRISPR/Cas9 applications in treatment of infectious disease

Virus type	Target gene	Cell/animal	Delivery method	Result	Ref.
HPV-16	E7	SiHa, Caski, C33A, and HEK293 cell lines	Plasmid	Induction of apoptosis and inhibition of tumor cell growth	[276]
HPV-16	E7	Mice	PEGylated liposome	Elimination of established tumors in immunocompetent mice	[277]
HIV-1	LTR	Jurkat cells and HeLa cell line	Plasmid	Efficient cleavage of LTR target sites	[278]
HPV-16	E6, E7	Mice	Plasmid	Activation of p53 and pRB signaling pathways, leading to impaired tumor growth	[279]
HBV	Various sites	Huh-7 cell line Mice	Plasmid	Clearance of intrahepatic HBV templates in vivo	[280]
HPV-16	E6, E7	SiHa and C33-A cell lines Mice	Plasmids Lipofectamine	Upregulation of p53 and p21 expression, leading to reduced tumor growth	[281]
HBV	Pcsk9	HEK293T cell line Mice	AAV	Reducing the HBV viral loads	[282]
HIV-1	LTR U3, T, and R region	HEK293T cell line	Lentivirus	Enabling prolonged adaptive defense versus new viral infection	[283]
HBV	Various sites	HEK293T-C, -Pol, and -S cell lines	Lipofectamine 3000 Lentivirus	Inhibition of viral gene expression	[284]
HPV-16	E7	SiHa and Hela cell lines, mice	Plasmids	Inhibition of tumor growth in nude mice	[285]
HIV-1	LTR U3 region	MEFs Mice and Rats	Lentivirus	Attenuation of HIV-1 replication	[286]
HIV-1	CCR5	HEK293T cells, TZM.bl cells, and CEMss-CCR5 cells	Lentivirus	CCR5 KO cells showed remarkable resistance to R5-tropic HIV-1	[287]
HPV-18	E6, E7	HeLa cell lines	Plasmids	Induction of pRb/p21 pathway resulted in senescence	[288]
HPV-16	E6, E7	Mice	AAV	Robust and selective decrease in tumor growth	[289]
HIV-1	CXCR4	Ghost-CXCR4 cells, Jurkat cells, and primary human CD4+ T cells	Lentivirus	Resistance to HIV infection	[51]
HPV-18	E6, E7	HeLa cell line	Plasmid	Reduced cancer cell proliferation	[290]
HIV-1	LTR	Latent microglial cells	Magnetic delivery	Deterring the latent HIV-1 infection in	[291]
HPV-16	E6/E7	SiHa cell line	Lipofectamine	Synergistic antitumor effect of E6/E7 KO using CRISPR system with PD1 inhibitors of cancer cell	[63]
HPV-18	E7	Hela cell line Mice	Micelle delivery, Lipofectamine	Reducing the HPV-induced cancerous activity	[292]
HIV-1	CCR5	iPSCs	PiggyBac transposon vectors	Resistance to HIV infection	[293]
HIV-1	LTR	HEK293T TZM-bl cells	Plasmid	Suppressing HIV-1 replication	[294]
HIV-1	CXCR4	TZM-bl cells	Lipofectamine 2000	Reduced HIV-1 replication	[295]
HPV-18	E6	HeLa, HCS-2, and SKG-I cell lines Mice	AAV	Improvement of p53 expression, leading to induction of apoptosis and negative regulation of tumor growth	[296]

Human papillomavirus (HPV)-16 and -18, human immunodeficiency virus (HIV)-1, hepatitis B virus (HBV), long terminal repeat (LTR), proprotein convertase subtilisin/kexin type 9 (PCSK9), C–C chemokine receptor type 5 (CCR5), C-X-C motif chemokine receptor 4 (CXCR4), mouse embryonic fibroblast (MEF), induced pluripotent stem cells (iPSCs), adeno-associated viral (AAV) vectors, retinoblastoma protein (pRB), programmed cell death 1 (PD-1)

The most promising editing targets of CRISPR/Cas9 therapy to combat HIV viruses are the C–C chemokine receptor 5 (CCR5) gene, C–C–C chemokine receptor 4 (CXCR4) gene, proviral DNA-encoding viral proteins, and the HIV 5′ and 3′ long terminal repeat (LTR) [47–49]. For instance, Ebina et al. (2013) showed the extraordinary capacity of the CRISPR/Cas9 machinery to affect the HIV-1 genome and avert its expression [50]. They found that LTR-targeting CRISPR/Cas9 reagents suppressed LTR-driven expression in HIV-1-infected T cells and cleaved and mutated LTR target sites, leading to perturbation of latent HIV-1 provirus [50]. Also, Cas9-induced ablation of CXCR4 in T cells resulted in their robust resistance to HIV without significant off-target effects and disrupting cell biological processes such as proliferation [51]. In addition to non-carcinogenic viruses (e.g., HIV), the CRISPR/Cas9 system offers the opportunity to modify the pathogenic process of carcinogenic viruses such as HPV and HBV. Viruses are causal agents of about 10–15% of all cancers worldwide in addition to their prominent role in infectious diseases. Among viruses, several DNA viruses, including Kaposi’s sarcoma herpesvirus (KSHV), Epstein–Barr virus (EBV), HPV, HBV, and simian virus 40 (SV40), along with two RNA viruses, viz. human T-lymphotropic virus-1 (HTLV-1) and hepatitis C virus (HCV), are the most well-defined carcinogenic viruses [52, 53]. Zhen and colleagues (2015) suggested that CRISPR/Cas9-mediated ablation of the surface antigen (HBsAg)-encoding region of HBV prohibited HBV replication in liver-derived cell lines, HepG2, and BALB/c nude mice, as evidenced by reduced levels of HBsAg secretion in cell culture and mouse serum [54]. Likewise, the CRISPR/Cas9 system targeted HBV covalently closed circular DNA (cccDNA) and inhibited HBV replication in HBV-infected Huh7 and HepG2 cells. By means of the CRISPR/Cas9 system, clearance of viruses from infected cells becomes hypothetically practical for any DNA- or RNA-mediated virus during their pathological process [55]. Thus, the CRISPR/Cas9 system may serve as a unique avenue for HBV therapy. In addition to the use of the CRISPR/Cas9 system alone, combination therapy of CRISPR/Cas9 with other modalities, such as the NU7026 P inhibitor, could efficiently eliminate the HBV genome from infected cells [56]. NU7026 P is a well-known suppressor of NHEJ and constrains CRISPR/Cas9-mediated degradation of cccDNA and results in large on-target deletions [56]. Thus, negative regulation of its activation may potentiate the efficacy of CRISPR/Cas9-mediated degradation of cccDNA, culminating in HBV genome eradication. Given the central role of early genes E6 and E7 in continuing the malignant phenotype of cervical cancer cells following HPV infection, the CRISPR/Cas system has recently been applied to target HPV16/18-E6 and-E7 DNA in HPV-infected cells [57, 58]. In this regard, HPV16-E7 ablation using the CRISPR/Cas system induced apoptosis and disrupted proliferation of cervical cancer SiHa and Caski cell lines in vitro with no effect on HPV-negative cells [59]. The E7 DNA deficiency resulted in upregulation of tumor suppressor protein retinoblastoma (pRb), suggesting E7 as a potential target for gene editing approaches to treat cervical cancer [59]. Furthermore, it has been shown that addition of the CRISPR/Cas9 system to immune checkpoint inhibitors (ICIs), as Food and Drug Administration (FDA)-approved anticancer drugs, may augment their antitumor effects [60–62]. For example, combination therapy using CRISPR/Cas9-mediated disruption of HPV16 E6/E7 gene and PD1 inhibitor resulted in an improved overall survival (OS) rate accompanied by impaired tumor development in SiHa tumor cell-bearing SCID mice [63, 64]. Also, cotreatment inspired the population of antigen-presenting cells (APCs), CD8+ and CD4+ T lymphocyte cells in tumor tissue, thereby eliciting robust antitumor responses in treated mice against tumor tissue [63].

A growing body of evidence indicates that the CRISPR/Cas9-mediated genetic targeting tool could be an alternative means to treat virus-related diseases in the future. Nonetheless, viruses can evade CISPR/Cas9-mediated inhibition by attaining several mutations at the target region, which disfavors gRNA interaction with the corresponding sequence, without deterring viral replication [65, 66]. Circumventing this drawback is thus urgently required before their wide application in the clinic.

### Genetic disorders

Gene targeting systems have provided a quick and operational means to target and modify the genome at specific sites. Many genes contribute to the pathogenesis of genetic disorders [67, 68]. Given that one particular genetic mutation causes such genetic disorders, the CRISPR/Cas9 machinery can be used to treat such disorders by targeting and modifying a single gene [69, 70]. Such targeting of genes can be accomplished both ex vivo and in vivo [71]. The target cells with mutated genes are isolated then manipulated by programmable nucleases to correct the mutated gene, and ultimately injected into the original host ex vivo [72, 73]. Engineered nucleases accompanied with the correct sequence of the target gene can be injected directly into the patient for systemic or targeted tissue (such as the eye, brain, or muscle) in vivo [74, 75]. During the recent decade, CRISPR/Cas9 has exhibited promising preliminary capability to treat β-thalassemia [76–78], tyrosinemia [79], Duchenne muscular dystrophy (DMD) [80, 81], hemophilia [82, 83], cystic fibrosis [84], central nervous system (CNS)-associated diseases [85, 86], Tay–Sachs diseases (TSD) [87], and fragile X syndrome disorders (FXS) [88, 89]. Indeed, this technology has enabled the correction of the multiple mutated genes associated with responding genetic disorders, including the DMD gene in DMD, CFTR gene in CF, factor IX gene in hemophilia B, hemoglobin beta-chain gene in β-thalassemia, presenilin 1 and 2 (PSEN1 and PSEN2) and apolipoprotein E4 (apoE4) genes in AD, HTT gene in HD, leucine-rich repeat kinase 2 (LRRKK2) gene in PD, fumarylacetoacetate hydrolase (FAH) in tyrosinemia, Hex gene in TSD, fragile X mental retardation 1 (FMR1) gene in FXS, etc. [90, 91].

A complete review of such CRISPR/Cas9 applications lies beyond the scope of this article, so the reader is referred to excellent articles in this field [92–94].

## CRISPR/Cas9 in cancers

**Fig. 2:**
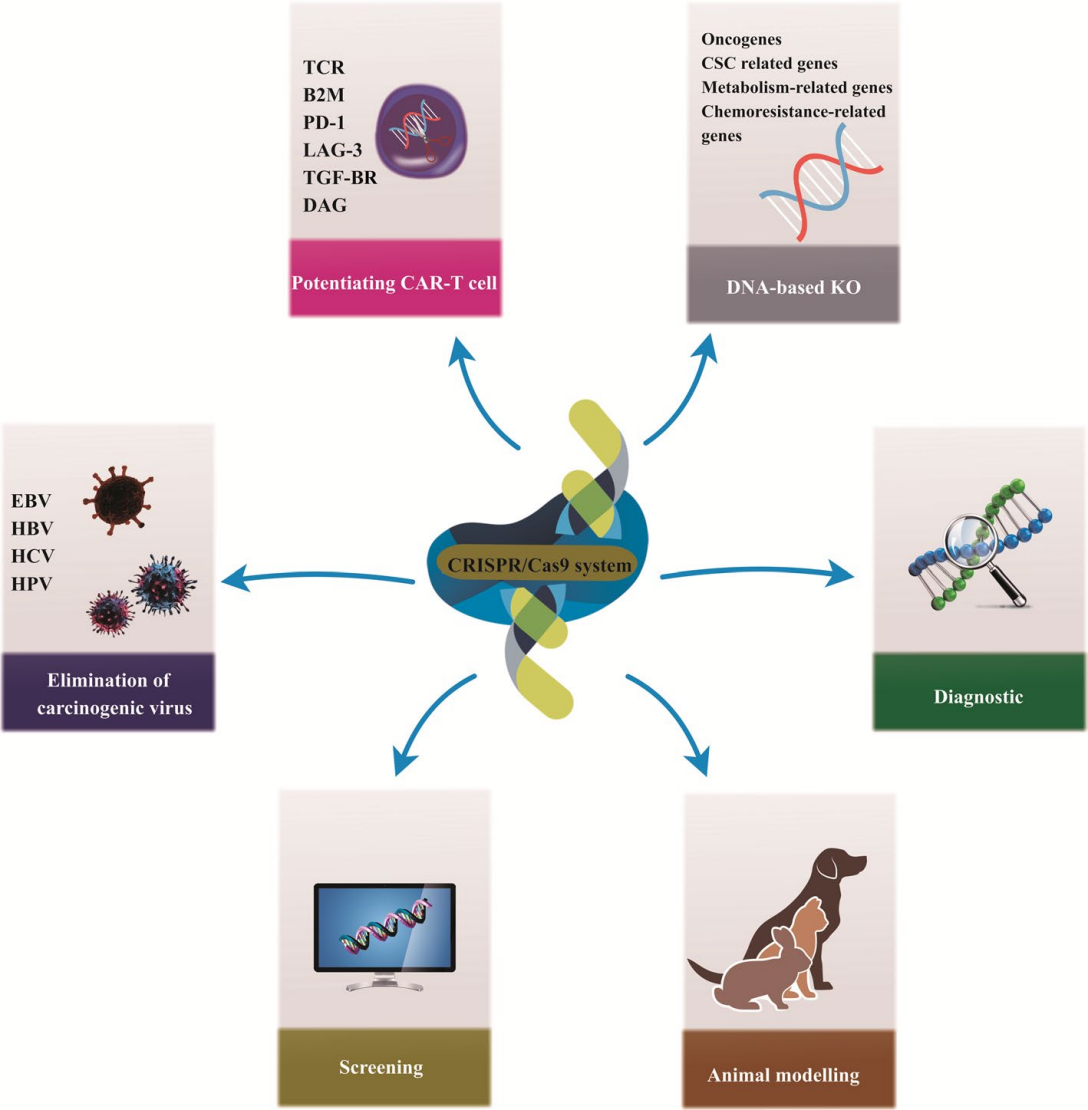
CRISPR/Cas9 applications in cancer research and therapy. Knockout (KO), T-cell receptor (TCR), chimeric antigen receptor (CAR)-T cell, β2-microglobulin (B2M), programmed cell death protein 1 (PD1 or PDCD1), lymphocyte activating gene 3 (LAG-3), transforming growth factor-beta receptor (TGF-βR), diacylglycerol (DAG), Epstein–Barr virus (EBV), human papillomavirus (HPV), hepatitis B virus (HBV), hepatitis C virus (HCV), cancer stem cell (CSC)

CRISPR/Cas9 tools have great capacity for the diagnosis and treatment of cancer, including (1) the use of CRISPR/Cas9-based diagnostic systems SHERLOCK and DETECTR for cancer diagnostics, (2) providing TCR knockout (KO) CAR-T cells (universal CAR-T cells), (3) KO of inhibitory receptors such as PD-1 and LAG-3 to promote the capability of cancer immunotherapy, (4) elimination of oncogenic virus-like HPV, (5) and establishment of in vivo tumor models by eliciting mutations in several genes [7, 45, 95–97] (Fig. 2).

In this section, we focus on the therapeutic potential of the CRISPR/cas9 system in cancer treatment (Table 3).

**Table 3:** CRISPR/Cas9 applications in cancer treatment

Cancer	Target gene	KO/KI	Cell line/animal	Delivery method	Result	Ref.
HCC	*NSD1*	KO	Huh7, Hep3B, SMMC-7721, HepG2, and SK-Hep1 cell lines Mice	Lentivirus	Inhibition of tumor progress by downregulation of NSD1/H3/Wnt10b signaling pathway	[98]
HCC	*PHGDH*	KO	MHCC97L cell line	Lentivirus	Induction of tumor cell death by improving ROS level	[114]
HCC	*LIN28B-AS1*	KO	HepG2 cell line Mice	Lipofectamine 2000	Attenuation of tumor growth	[106]
HCC	*G9a*	KO	HepG2, Hep3B, SMMC-7721, BEL7402, and MHCC97L cell lines Mice	Lentivirus	Suppression of cell proliferation and metastasis both in vitro and in vivo	[297]
HCC	*HBsAg*	KO	LC/PRF/5, HepG2‐2.15, Hep3B, SK‐hep1, HLF, and Huh‐7 Mice	Lentivirus	Reducing HBsAg expression and inhibiting cell proliferation and tumorigenicity	[298]
HCC	*ANGPT2*	KO	Hep3B, SNU182, SNU387, and Li7 cell lines Mice	Lentivirus	Reduced angiogenesis	[109]
CRC	*CCAT1*	KO	HT-29 and SW-480	Plasmid (pX459, pX460-1, pX461-1)	Lowering the anchorage-independent growth	[115]
CRC	*MUC5AC*	KO	HCT-8 and LS174T cell lines Mice	Lipofectamine 2000	Reducing tumorigenesis and chemoresistance by targeting CD44/β-catenin/p53/p21 signaling	[121]
CRC	*DACH1*	KO	HCT116 and SW620 cell lines	Lentivirus	Decreasing organoid formation and size	[127]
CRC	*Par3L*	KO	CaCO-2 cell lines	Plasmid	Attenuation of proliferation and increasing cell apoptosis by caspase-3 activation Enhanced susceptibility to chemotherapy and radiotherapy	[123]
CRC	*LSD1*	KO	T29, SW480, HCT116, LoVo, and RKO cell lines	Lentivirus	Inhibition of Akt-mediated EMT and migration	[129]
CRC	*PP2A*	KO	HCT-116 and HT-29 lines Mice	Lentivirus	Inducing AMPK signaling to avert cell proliferation	[299]
BC	*FASN*	KO	MCF-7 cell line	Plasmid (px459)	Reducing cell proliferation, migration, and viability	[140]
BC	miR-23b and miR-27b	KO	MCF-7 cell line Mice	Lentivirus	Impaired tumor growth	[300]
CRC BC	*GPVI*	KO	MC38 and MC38-CEA colon and AT3 and E0771 breast cancer cell lines Mice	Lentivirus	Inhibition of tumor metastasis	[144]
BC	Osteopontin	KO	MDA-MB-231 cell line	Lentivirus	Eliciting significant radiosensitivity	[151]
BC	*FUT8*	KO	MCF-10A, MDA-MB-231, Hs578T, and T-47D cell lines Mice	Plasmid	Inhibition of invasive ability of cancer cell	[146]
BC	*RLIP*	KO	MCF7, MCF-10A, and MDA-MB231 cell line	Lentivirus	Hindrance of cell proliferation	[301]
BC	*DCLK1*	KO	BT474 and T47D cell lines	Lentivirus	Inhibition of cell metastasis	[148]
BC	PTPN23	KO	BT474, Cal51, MDA-MB-231, and MDA-MB-468 cell lines	Lentivirus	Reduced tumor outgrowth	[302]
BC	*CDK8*	KO	MCF7 and BT474 cell lines	Lentivirus	Suppression of ER-positive breast cancer cell proliferation	[303]
Cervical cancer	*AKR1B1*	KO	HeLa cell line	Lentivirus	Inhibition of cancer cell growth	[159]
Cervical cancer	*CD109*	KO	C33A, C4-1, CaSki, and SiHa cell lines	Lipofectamine	Negative regulation of cancer cell migration and proliferation by downregulation of EGFR-mediated STAT3	[161]
Cervical cancer	*IER5*	KO	Siha and Hela cell lines	Lentivirus	Eliciting radiosensitivity	[304]
Cervical cancer	Myostatin	KO	HeLa cell line	Lentivirus	Induction of apoptosis, ROS generation, and promoting fatty acid oxidation	[164]
SCLC	*MYCN*	KO	Mice	Lentivirus	Reducing chemoresistance by constraining USP7 activity	[174]
NSCLC	*YES1*	KO	H1792, H2009, and A549 cell lines Mice	Lentivirus	Inhibition of tumor growth and metastasis by suppression of mTOR	[168]
Various cancers	*PRMT5*	KO	H2171, A549, MiaPaCa2, A172, and MCF-7 cell lines	Lentivirus	Enhancing cell susceptibility to PRMT5 inhibition	[305]
NSCLC	*FRK*	KO	H292, H460, and H446 cell lines Mice	Lentivirus	Reducing cell proliferation, invasion, colony formation, and EMT process	[171]
GC	*PDEF*	KO	AGS cell line	Plasmid (pX459)	Inhibition of cell migration and invasion	[211]
GC	*METTL3*	KO	AGS cell line	Lipofectamine	Inhibition of cell proliferation	[306]
GC	*BCAM*	KO	BGC-823 and SGC-7901 cell lines	Lipofectamine	Inhibition of cell invasion and metastasis	[307]
Pancreatic cancer	*HIF-1α*	KO	BxPC-3 cell lines	Plasmids	Inhibition of metastasis by downregulation of VEGF and MMP-9	[184]
Pancreatic cancer	*ATG12*	KO	MIA PaCa-2 and AR42J cell lines	Plasmid (pX458)	Inhibition of pancreatitis-induced autophagy	[186]
Various cancers	*EI24*	KO	MIA PaCa-2, Panc-1, HeLa, and U2OS cell lines	Lentivirus	Diminished autophagy	[187]
PDAC	*MUC16*	KO	Capan-1 and Colo-357 cell lines	Retrovirus	Reducing tumor-associated carbohydrate antigens	[308]
Various cancers	*FOs*	KO	A673, RD-ES, and U2OS cell lines mICE	Lentivirus	Lowering tumor burden/mortality	[192]
Neuroblastoma	*MYCN*	KO	Various cell lines Mice	Lentivirus	Deterring neuroblastoma growth	[309]
EC	*ETV4*	KO	T-47D cell line Mice	Plasmid	Reduced tumor cell growth	[310]
EC	*PTEN*	KO	Ishikawa, AN3CA, Nou-1, Hec-108, and Hec-1A cell lines Mice	Lentivirus	Reduced tumor cell growth	[311]
NPC	*SRPK1* and *SRPK2*	KO	CNE1 cell line	Plasmid	–	[312]
Melanoma	*CDK2*	KO	A375 cell line	Lentivirus	Induction of G0/G1 phase arrest and apoptosis	[313]
EOC	*BMI1*	KO	SKOV3 cell line Mice	Plasmid (pX330)	Inhibition of tumor cell growth and metastasis, promoting cell apoptosis, and enhancing platinum sensitivity	[314]
Bladder cancer	*UCA1*	KO	5637 and T24 cell lines Mice	Plasmid	Robust suppression of cell proliferation, migration, and invasion	[315]
ATC	*EGFR*	KO	SW579 cell line	Plasmid	Inducing cell cycle arrest, inhibition of cell growth, and metastasis	[316]
Prostate cancer	*GPRC6A*	KO	PC-3, DU145, LNCap, and 22Rv1 cell lines Mice	Lentivirus	Impaired tumorigenesis	[195]
Prostate cancer	*BRCA2*	KO	LNCaP, DU145, 22RV1, and TRAMP-C2 cell lines Mice	Lentivirus	Antiproliferative effects	[196]
Prostate cancer	*Akt1*/*2*	KO	CWR22Rv1 cell line Mice	Plasmid (px300)	Suppressed metastasis	[197]

Hepatocellular carcinoma (HCC), colorectal cancer (CRC), breast cancer (BC), small cell lung cancer (SCLC), non-small cell lung carcinoma (NSCLC), pancreatic ductal adenocarcinoma (PDAC), endometrial cancer (EC), nasopharyngeal carcinoma (NPC), epithelial ovarian cancer (EOC), nuclear receptor binding SET domain-containing protein 1 (NSD1), phosphoglycerate dehydrogenase (PHGDH), hepatitis B surface antigen (HBsAg), angiopoietin 2 (ANGPT-2), colon cancer associated transcript 1 (CCAT1), mucin 5AC (MUC5AC), Dachshund homolog 1 (DACH1), partitioning defective 3-like protein (Par3L), lysine-specific demethylase 1 (LSD1), protein phosphatase 2A (PP2A), fatty acid synthase (FASN), glycoprotein VI (GPVI), fucosyltransferase 8 (FUT8), Ral-interacting protein (RLIP), Doublecortin-like kinase 1 (DCLK1), protein tyrosine phosphatase non-receptor type 23 (PTPN23), cyclin-dependent kinase 8 (CDK8), aldo–keto reductase family 1, member B1 (AKR1B1), immediate-early response 5 (IER5), protein arginine methyltransferase 5 (PRMT5), Fyn-related Src family tyrosine kinase (FRK), prostate-derived ETS factor (PDEF), methyltransferase 3, *N*^6^-adenosine methyltransferase complex catalytic subunit (METTL3), basal cell adhesion molecule (BCAM), hypoxia-inducible factor (HIF)-1, autophagy related 12 (ATG12), mucin 16 (MUC16), ETS variant transcription factor 4 (ETV4), phosphatase and TENsin homolog deleted on chromosome 10 (PTEN), serine/arginine-rich protein-specific kinase (SRPK) 1/2, cyclin dependent kinase 2 (CDK2), urothelial cancer associated 1 (UCA1), epidermal growth factor receptor (EGFR), G protein-coupled receptor family C group 6 member A (GPRC6A), knockout (KO), knock-in (KI), reactive oxygen species (ROS), epithelial–mesenchymal transition (EMT), AMP-activated protein kinase (AMPK), estrogen receptor (ER), vascular endothelial growth factor (VEGF), matrix metalloproteinase-9 (MMP-9)

### Liver cancer

Recently, targeting of various genes in liver cancer cells using the CRISPR/Cas9 system has demonstrated a potential ability to impair their proliferation and metastasis. In 2019, Zhang and colleagues designed a specific sgRNA to target nuclear receptor-binding SET domain-containing protein 1 (NSD1) in HCC cell lines [98]. The NSD1 histone lysine methyltransferase targets the Wnt/β-catenin signaling pathway associated with HCC tumorigenesis. They found that CRISPR/Css9-mediated NSD1 KO HCC cells displayed reduced proliferation, migration, and invasion in vitro and in vivo [98]. NSD1 ablation brought about improved methylation of H3K27me3 and reduced methylation of H3K36me2, leading to downregulation of Wnt10b expression. Therefore, the CRISPR/Cas9 tool may hinder HCC oncological events by negatively regulating the Wnt/β-catenin signaling axis in nude mice and in vitro [98]. In HCC, targeting the Wnt/β-catenin signaling axis using CRISPR/Cas9 machinery could exert a positive antitumor effect in HEK 293T cell line, as evidenced by their perturbed proliferation [99]. Likewise, CRISPR/Cas9-mediated ablation of acid-sensing ion channels 1a (ASIC1a), which triggers migration and invasion in liver cancer, could deter cell proliferation and tumorigenicity [100]. The empirical consequences are caused mainly by stimulation of β-catenin degradation and coactive lymphoid enhancer factor/T cell factor (LEF/TCF) inactivation in HCC cell lines and also xenograft mice following ASIC1a ablation [100]. Mechanistically, the β-catenin inspires downstream signaling transduction by LEF-TCF, which eventually induces c-MYC expression [101]. In malignant cells, the β-catenin/LEF/TCF axis is often prompted and triggers cell proliferation [102]. Besides, inhibition of this pathway may offer great potential to moderate HCC proliferation, migration, and invasiveness [103]. Also, dysregulation of insulin-like growth factor 2 (IGF2) mRNA-binding protein 1 (IGF2BP1) has been suggested to be involved in HCC progression [104]. IGF2BP1 is required to stabilize and translate various oncogenes, such as glioma-associated oncogene homolog 1 (*Gli1*) and *Myc 91*, thus its expression is associated with worse prognosis in HCC patients [104, 105]. LIN28B-AS1 directly binds to IGF2BP1 like long non-coding RNAs. Zhang et al. (2020) designed specific sgRNA targeting and modified LIN28B-AS1 expression to evaluate LIN28B-AS1 ablation on HCC proliferation pathological events [106]. They found that IGF2, Gli1, and Myc expression was substantially downregulated in LIN28B-AS1-deficient HCC cell lines in vitro by reducing IGF2BP1 mRNA levels, suppressing HCC cell proliferation and invasion [106]. In nude mice, LIN28B-AS1 KO HepG2 xenograft tumors had a slightly increasing trend compared with normal LIN28B-AS1-positive HepG2 xenograft tumors [106]. In addition to gene editing tools, negative regulation of IGF2BP1 synthesis in HCC cells using specific siRNA dissuades tumor proliferation and invasion [107]. These findings make IGF2BP1 a potent target for HCC therapy with the aim of delivering novel therapeutic plans with improved efficacy.

Angiogenesis plays a fundamental role in tumor progression. Meanwhile, the angiopoietin-2 (ANGPT2)/Tie2 pathway induces angiogenesis in HCC tumors by directly targeting the proliferation of endothelial cells [108, 109]. Accordingly, the ANGPT2/Tie2 axis has been suggested as a reasonable target for antiangiogenic therapy. Targeting ANGPT2 is presently undergoing phase II clinical trials, with preliminary results suggesting encouraging antitumor activity and safety [110]. In 2020, Xie and colleagues showed that CRISPR/Cas9-mediated ablation of ANGPT2 in Hep3B and MHCC97H cell lines diminished the potential of their derivative exosome to promote proliferation of ECs [109]. It thus appears that this pathway could be a putative therapeutic target for antiangiogenic treatments. Also, the role of miR-3188 in HCC pathogenesis has recently been manifested, where its overexpression improves cell viability and proliferation but suppresses apoptosis of HCC cells [111, 112]. Meanwhile, Zhou et al. (2017) showed that miR-3188 ablation could constrain cell growth and colony formation, induce cell cycle arrest (G0/G1 phase), and instigate apoptosis in HepG2 cells [113]. miR-3188 inactivation could also diminish migration and invasion due to downregulation of Notch1 activation in HCC cells [113].

Importantly, gene editing tools can defeat HCC resistance to conventional treatments such as sorafenib therapy. Because of the central role of phosphoglycerate dehydrogenase (PHGDH), which serves a critical role in serine synthesis and triggering HCC resistance to sorafenib, Wei et al. (2019) highlighted its potency to compromise Sorafenib resistance [114]. They showed that downregulation of nicotinamide adenine dinucleotide phosphate (NADPH) enforced PHGDH KO HCC cells to increase reactive oxygen species (ROS) levels. Manipulated cells also showed higher apoptosis rates upon sorafenib treatment than nonmanipulated cells [114]. It was proposed that PHGDH ablation results in negative regulation of the synthesis of antioxidant mediators (e.g., NADPH) and then makes PHGDH KO HCC cells susceptible to sorafenib [114].

### Colorectal cancer (CRC)

The latest investigation has revealed that the CRISPR/Cas technique could target long non-coding RNAs (lncRNAs), thus enabling CRC treatment. Researchers have sought different strategies to suppress their activity to achieve better therapeutic outcomes. For instance, CRISPR/Cas9-mediated ablation of lncRNA CCAT1 gene in other CRC cell lines, SW-480 and 14 HCT-116, could impair their anchorage-independent growth [115]. CCAT1 expression has an intimate association with the CRC stage and stimulates cell growth and mobility by targeting miR-181a-5p [116]. Therefore, it may be possible to target CRC therapy due to its undesired biological activities. Likewise, in mouse and human tumor-derived organoids, simultaneous targeting of adenomatous polyposis coli (APC) and KRAS, which mainly contribute to the disease progress in the early stage of CRC, brought about robust antitumor effects [117]. In addition, secretory mucin (MUC) 5AC has recently been suggested as a putative target for targeted therapy [118, 119]. MUC5AC is a large gel-forming glycoprotein expressed aberrantly during CRC stages [120]. A recent study in subcutaneous and colon orthotopic mouse models demonstrated that MUC5AC-deficient CRC cells possess less tumorigenic capacity [121]. Also, MUC5AC-deficient tumor-cell-bearing mice exhibit reduced appearance of metastatic lesions [121]. Since MUC5AC induces chemical resistance through CR44/β-catenin/p53/p21 signaling in CRC 107, combination therapy with gene editing tools and chemotherapeutic agents can break CRC resistance to conventional chemotherapies [121]. In addition to MUC5AC, it has been proposed that ablation of partitioning defective 3-like protein (Par3L) 108, a recently described cell polarity protein, and nuclear factor-erythroid factor 2-related factor 2 (Nrf2) [122], a critical transcription factor, may attenuate CRC cell resistance to chemotherapies and irradiation. Mechanistically, Par3L plays a crucial role in CRC survival via negative regulation of the liver kinase B1 (LKB1) Lkb/AMP-activated protein kinase (AMPK) signaling pathway [123]. Besides, NRF2 potentiates amino acid and protein synthesis in CRC cells [122], so targeting its expression could result in encouraging outcomes in CRC. Another study applied the CRISPR/Cas9 technique to target dachshund homolog 1 (DACH1), a target expressed explicitly in discrete crypt base cells [124]. DACH1 protein promotes tumorigenesis, invasion, and metastasis by deregulating the bone morphogenetic protein (BMP) signaling pathway [125, 126]. Importantly, its levels are usually found to be boosted in all stages of CRC [127]. Nonetheless, KO of DACH1 expression using CRISPR technique and shRNA could deter CRC cell growth, attenuate organoid formation efficiency, and organoid tumor size [127]. These results shed light on the role of DACH1 and introduce a possible prognostic marker and therapeutic goal for CRC patients [127]. Furthermore, lysine-specific demethylase 1 (LSD1), a well-known chromatin-modifying enzyme, is overexpressed in CRC and associated with proliferation and migration mainly by transduction of the phosphoinositide 3-kinase (PI3K)/Akt axis [128]. In this regard, Miller et al. applied specific sgRNA to block its expression in CRC cell lines and showed that CRISPR/Cas9-mediated LSD1 ablation corresponded to inhibition of AKT-induced epithelial–mesenchymal transition (EMT) and migration [129]. Other studies have outlined that inactivation of LSD1 by gene editing techniques could inhibit the proliferation and migration of leukemia [130, 131], Merkel cell carcinoma (MCC) [132], and HCC cells [133].

### Breast cancer

It has been strongly evidenced that altered expression of miRNAs is involved in breast cancer progression [134, 135]. In this regard, miR-23b and miR-27b promote tumor progress in various human tumors and may provoke the angiogenesis process in this setting. Recent studies in MCF7 breast cancer cells demonstrated that KO of miR-23b and miR-27b gene expression using CRISPR systems alleviated tumor growth in xenograft nude mice by upregulation of ST14 (suppression of tumorigenicity 14) [136]. ST14 typically decreases breast cancer cell proliferation and invasion [137, 138], so antitumor effects upon inactivation of miR-23b and miR-27b may depend on promotion of ST14 activity. Increasing evidence also shows that dysregulated expression of fatty acid synthase (FASN), complicating the endogenous synthesis of fatty acids and the adjustment of ERα signaling, may contribute to breast cancer onset and progress [139]. In 2020, Gonzalez-Salinas et al. showed that CRISPR/Cas9-mediated genetic depletion of FASN inhibits aggressive features in breast cancer MCF-7 cells, as verified by impaired cell proliferation, viability, and migration [140]. Importantly, transcriptomic studies have revealed that FASN deficiency has a more evident negative effect on proliferation-associated genes than lipid metabolism [140]. These results were also confirmed by analysis of the impact of FASN KO on oncogenic activities in leukemia cells [141].

Furthermore, targeting platelet glycoprotein VI (GPVI), which acts as a metastasis inducer by interaction with cancer cell-derived galectin-3, resulted in marked antitumor activities in vitro and in vivo [142]. GPVI causes the maintenance of tumor vessel integrity and mediates interactions between platelets and cancer cells. Platelets protect cancer cells from attack by natural killer cells (NKCs) [143], so perturbing platelet–cancer cell interaction may disrupt tumor cell progress. In this regard, Mammadova-Bach and coworkers (2020) reported that KO of platelet GPVI in mice led to a drop in breast cancer cell metastasis [144]. Also, GPVI inhibitors were found that could provide an obstacle to ovarian [142] and prostate [145] cancer metastasis. GPVI may thus be a potential target for antimetastatic treatments. Also, impaired breast cancer cell proliferation and metastasis were observed following CRISPR/Cas9-mediated inactivation of fucosyltransferase 8 (FUT8), a critical positive regulator of cell growth and tumor metastasis core fucosylation of target biomolecules [146]. FUT8 ablation alleviates TGF-β signaling and EMT in breast cancer by inhibiting TGF-β core fucosylation, disturbing breast cancer lung metastasis in mice xenografts [146].

Since increased expression of doublecortin-like kinase 1 (DCLK1) has been reported in patients with breast cancer associated with poor prognosis, targeting DCLK1 has been proposed as a possible candidate in the field of antitumor study [147]. Liu and coworkers (2019) found that DCLK1 KO in breast cancer cell line BT474 using CRISPR technology suppressed its metastatic features [148]. These beneficial effects were likely related to upregulation of tight junctions (TJ)-associated protein Zonula occludens (ZO-1) along with downregulation of zinc-finger E-box binding homeobox 1 (ZEB1), a master regulator of EMT [148]. Indeed, upregulation of TJ-associated protein expression and conversely suppressing ZEB1 activation, in turn, leads to reduced cell motility and invasiveness [148]. Gene editing tools can offer a practical possibility for overcoming cancer cell resistance to conventional therapies. Due to the proven role of the osteopontin (*OPN*) gene in inducing resistance to radiotherapy (RT) [149, 150], the impacts of its ablation in conjunction with RT have been highlighted [151]. Accordingly, Behbahani et al. (2021) indicated that the viability of the OPN-deficient breast cancer MDA-MB-231 cell line was severely reduced upon RT compared with the nonmanipulated MDA-MB-231 cell line [151]. It can thus be supposed that inactivation of the *OPN* gene might become an effective therapeutic plan to circumvent tumor cell resistance to conventional therapies, such as RT [151].

### Cervical cancer

Targeting oncoproteins E6 and E7 in HPV16 and HPV18 utilizing gene editing tools could inactivate such oncogenes and thus prompt cell cycle arrest and apoptosis [58, 152]. For example, Ling et al. (2020) showed that double-targeting of E6 and E7 improved p53 and p21 protein levels in cervical cancer lines (HeLa and SiHa) and tumor cell-bearing mice [58]. Given that HPV E6 stimulates inactivation of p53 in tumor cells, reactivation of its expression and transduction of p53 signaling upon E6 ablation has been recommended as a putative scheme for cervical cancer therapy [153]. In addition to the CRISPR system, ZFNs- [154] and TALEN-based [155] targeting of HPV16/18 E7 could efficiently block expression of E7 oncogenes and lead to apoptosis induction in HPV16 HPV18-infected cervical cancer cells.

The latest research has shown that targeting aldo–keto reductase family one member B1 (AKR1B1), which is highly expressed in several tumors and correlates with tumor growth, could benefit cervical cancer [156]. AKR1B1 contributes to prostaglandin F2α (PGF2α) synthesis and protein kinase C (PKC) transduction, which in turn triggers upregulation of NF-kB, inflammation, and inflammation proliferation [157]. Improved AKR1B1 levels and potentiated activity are usually detected in cervical cancer, which hypothetically correlates with higher prostaglandin E2 (PGE2), a well-known inducer of cervical carcinogenesis [156, 157]. The establishment of human endometrial KO cell lines using CRISPR/Cas9 technology confirms the PGs synthase function of AKR1B1 [158]. In vitro studies have shown that AKR1B1-deficient cervical cancer cell lines exhibit lower proliferation, migration, and invasion than nonmanipulated cells [159]. Concerning recent reports, AKR1B1 suppression could constrain PGE2 activity and thus disturb cervical carcinogenesis by preventing angiogenesis and cancer cell proliferation as well as inducing apoptosis [160]. Also, CD109, as a result of its role in transforming growth factor-β1 (TGF-β1) signaling and signal transducer and activator of transcription 3 (STAT3) activation, could be an innovative target for cervical cancer therapy [161–163]. CD109 is drastically expressed in cervical cancer and upregulates epidermal growth factor receptor (EGFR)-mediated STAT3 phosphorylation, enabling cervical cancer cell migration and proliferation, and supporting cancer cell phenotype [161]. However, Mo et al. (2020) demonstrated that targeting CD109 by siRNA or CRISPR/Cas9 could inhibit cervical cancers’ tumorigenic and aggressive properties by inactivating the CD109/EGFR/STAT3 axis in vitro and in vivo [161].

Furthermore, KO of growth differentiation factor-8 (GDF-8), or myostatin, a protein that is highly overexpressed in human tumors, by using the CRISPR/Cas9 technique could induce apoptosis intrinsic pathways in HeLa cells and prohibit their proliferation [164]. The observed effects are probably caused by increased ROS intracellular levels and promotion of elevated fatty acid oxidation, which leads to induction of mitochondrial membrane depolarization, secretion of cytochrome c (Cyt-c), and finally induction of the caspase cascade [164]. Similarly, targeting GDF-8 expression in Lewis lung carcinoma (LLC) cells impaired their proliferation and growth in vitro and in vivo [165]. Also, KO of GDF-8 promotes skeletal muscle mass in tumor-bearing rodents through upregulation of the Akt/mTOR pathway, easing the production of skeletal muscle proteins [165].

### Lung cancer

Recent studies have highlighted the role of YES1 in lung cancer development, identifying YES1 as a potential target involved in lung cancer carcinogenesis [166]. YES1 adjusts cell growth, survival, apoptosis, cell–cell adhesion, and cytoskeleton remodeling. Its levels have been found to be enhanced in patients with lung cancer, making it a potential therapeutic target in lung cancer [167]. The vital role of YES1 in lung carcinogenesis was revealed by its obstruction using the CRISPR/Cas9 system, leading to disrupted growth and metastasis of NSCLC by downregulation of mTOR signaling, a positive regulator of carcinogenesis [168]. Also, genetic depletion of YES1 made dasatinib-resistant NSCLC cell lines susceptible to dasatinib-induced antitumor effects in vitro [168]. Its congenital absence also led to promising impacts in other malignancies, such as breast [169] and ovarian cancers [170]. Moreover, Zhang et al. (2020) evaluated the possible effect of the genetic depletion of Fyn-related kinase (FRK) by CRISPR/Cas9 in lung carcinoma H1299 cells to elucidate its role in NSCLC pathogenesis [171]. FRK potentiates the stemness phenotype of NSCLC and triggers the EMT process by eliciting metabolic reprogramming [172, 173].

Interestingly, FRK depletion impaired the stemness phenotype of H1299 by downregulation of CD44 and CD133 expression and concurrently stimulated metabolism reprogramming by blocking the Warburg effect and varying the energy type in H1299 cells [171]. Also, FRK-deficient H1299 cells demonstrated attenuated proliferation, invasion, colony formation, and EMT process in vitro. These findings indicate that FRK could be a putative target for lung carcinoma therapy [171].

In 2020, Grunblatt and colleagues showed that CRISPR/Cas9-mediated KO of oncogene *N-MYC* may yield small cell lung cancer (SCLC) [174]. Amplification of N-MYC is a well-recognized poor prognostic marker for human tumors and is associated with aggressive tumor features and resistance to conventional therapies [175]. The results of a study conducted in chemosensitive patient-derived xenograft (PDX) models of SCLC revealed that inactivation of N-MYC restores cancer cell chemosensitivity through downregulation of ubiquitin-specific protease 7 (USP7) expression [174]. USP7 favors DNA damage response and stimulates cancer progress by negative regulation of p53, and is associated with poor survival rate in cancer patients [176, 177]. Hence, inactivating its expression using inhibition of N-MYC expression or its direct ablation has been an imperative strategy in cancer therapy [176, 178].

### Pancreatic cancer

*KRAS* mutation has been confirmed as the primary contributor to pancreatic cancer carcinogenesis, being mutated in ~ 95% of pancreatic neoplasias [179]. In 2019, Lentsch et al. found that efficient KO of c.35G > A (p.G12D) *Kras* mutation in human pancreatic cancer cell lines SUIT-2 and Panc-1 and mouse cell lines TB32047 is possible [180]. Studies in pancreatic ductal adenocarcinoma (PDA) rodent models indicated that KRAS favors immune escape in pancreatic cancer cell-bearing mice by activating the BRAF and MYC axis [181]. However, KRAS genetic depletion using the CRISPR system provokes an antitumor response against PDA cells. Of course, KRAS ablation attenuates, but does not eliminate, the tumorigenic potential of PDAC cells, suggesting that the multifaceted axis complicates the progress of PDA [181]. Given that the hypoxic tumor microenvironment (TME) supports the growth and metastasis of pancreatic cancer cells [182], inactivation of hypoxia-inducible factor-1α (HIF-1α) with CRISPR/Cas9 is suggested as another rational therapeutic approach [183]. For the first time, Li et al. (2019) developed a tumor-targeted lipid-based CRISPR/Cas9 delivery system to inhibit HIF-1α expression in vitro and in vivo [184]. They showed that ablation of HIF-1α resulted in lower expression of its downstream targets such as vascular endothelial growth factor (VEGF) and matrix metalloproteinase-9 (MMP-9), ensuring reduced metastasis and ameliorating the paclitaxel-driven cytotoxicity on human pancreatic cancer cell line BxPC-3 in vitro and in vivo [184]. It appears that combining CRISPR technology with conventional therapies could be a more efficient antitumor strategy. Likewise, Wei and colleagues (2020) revealed that ablation of protein arginine methyltransferase 5 (PRMT5), a central transcriptional regulator, by using the CRISPR/Cas9 technique enhances the susceptibility of PDAC cells to gemcitabine by inducing cell cycle arrest [185]. Other studies have signified that targeting autophagy may affect aggressive features of pancreatic cancer [186]. Meanwhile, Hwang and coworkers (2019) evaluated the role of EI24 (etoposide-induced gene 2.4 kb; PIG8, p53-induced gene 8) as a component of autophagy in pancreatic cancer cell growth [187]. They found that knockdown (KD) or KO of EI24 utilizing siRNA or CRISPR/Cas9, respectively, impaired pancreatic cancer autophagy and then suppressed cell proliferation [187]. These results indicate that EI24 acts as a tumor inducer in pancreatic cancer cells; however, there are some conflicting reports. For example, Zang et al. (2018) described that EI24 inhibits cell proliferation and stimulates cell cycle arrest in PDAC cells by triggering autophagic lysosomal degradation of c-Myc proto-oncogene [188]. Therefore, further analysis of the data and execution of more comprehensive studies are required to clarify the detailed role of EI24 in pancreatic cancer carcinogenesis.

### Prostate cancer

Many studies have revealed that activating protein-1 (AP-1), a transcription factor, is related to cancer onset and progress [189]. The proto-oncogenes *JUN* and *FOS* are pivotal in prostate cancer progression and invasion [190]. In prostate cancer cells, Ouyang et al. (2008) showed that forced expression of c-Fos and c-Jun stimulates tumorigenicity and provokes transduction of ERK/MAPK signaling [191]. Besides, Riedel and coworkers (2021) evidenced that CRISPR/Cas9-mediated inactivation of Jun results in impaired prostate cancer cell proliferation and invasiveness in vitro and in vivo [192]. Also, ablation of FOS potentiates Jun expression, and CRISPR/Cas9-mediated KO of Jun constrains prostate cancer cell proliferation [193]. Hence, targeting AP-1 transcription factors in prostate cancer by genome edition could be a therapeutic approach. In addition to AP-1, G protein-coupled receptor family C group 6 member A (GPRC6A) as a functional osteocalcin and testosterone sensing receptor contributes to prostate cancer growth [194]. In this regard, its upregulation enables prostate cancer cells to grow in response to dietary and bone-derived ligands [194]. Although it induces the EMT process of prostate cancer, KD of GPRC6A attenuates such cell invasion [194]. Importantly, GPRC6A-deficient prostate cancer cell line PC-3 created by CRISPR/Cas9 technology demonstrates drastically lower growth and aggression than nonmanipulated cells in vitro and in vivo [195]. Also, manipulated cells showed reduced ligand-dependent responses in vitro due to downregulation of extracellular-signal-regulated kinase (ERK) activity [195]. In another study, Chakraborty et al. (2021) designed a specific sgRNA to target expression of BRCA2, a key component of DNA damage repair (DDR). Its mutations have a tight association with prostate cancer oncological events [196]. Variations in DDR pathway genes such as *BRCA1*/*2* and *ATM* occur in 20–25% of men with metastatic castration-resistant prostate cancer (mCRPC) and complicate cancer cell resistance to therapeutic modalities [196]. They found that genetic depletion of *BRCA2* established by the CRISPR system caused an antiproliferative effect on prostate cancer cells and enhanced their susceptibility to poly (ADP-ribose) polymerase (PARP) inhibitors, FDA-approved drugs for mCRPC treatment [196]. Finally, double KO of *Akt1* and *Akt2* genes potently decreased prostate cancer cell metastasis in vitro and in vivo [197]. Aberrant expression of Akt1 and Akt2 with poor prognosis is shown in various cancers, such as colon [198], gastric [199], breast [200, 201], NSCLC [202], ovarian [203], HCC [204], and pancreatic cancers [205]. Indeed, Akt promotes cell survival, metastasis, and angiogenesis by downregulation of proapoptotic signals, such as Bad and Forkhead box O (FOXO) transcription factors, and transducing VEGF signaling axis [206–208]. Interestingly, Su et al. (2021) exhibited that Akt1- and Akt2-deficient prostate cancer CWR22rv1 cells exhibited an enormous invasive reduction in vitro and in vivo [197]. Thereby, inactivation of its expression and activity could offer promising outcomes in vivo.

CRISPR/Cas9 application has also attracted increasing attention for treating other human cancers, such as gastric cancer and glioma [209, 210]. Zhang et al. (2019) recently showed that CRISPR/Cas9-mediated ablation of the prostate-derived Ets factor (*PDEF*) gene resulted in suppression of the migration and motility of human gastric cancer AGS cells [211]. PDEF as a member of the Ets family of transcription factors serves a key role in stimulating tumorigenesis in gastric cancer, and elevated levels of PDEF correlate with poor prognosis [211]. Thus, targeting its expression could be a putative therapeutic strategy to hinder gastric cancer cell proliferation and metastasis [211]. Likewise, targeting sodium/glucose cotransporters 1 (SGLT1) protein, primarily expressed in various human tumors, is an effective plan to moderate gastric cancer pathogenesis [212, 213]. Its expression is positively related to histological differentiation and worse overall survival in gastric cancer patients [214]. Accordingly, CRISPR/Cas9-mediated ablation of SGLT1 averts proliferation of gastric cancer cells, induces their apoptosis, and could thus modify the metabolism of gastric cancer cells [214]. These results make it a rational target to control the development of gastric cancer cells by influencing their key oncogenic activities. In addition, Haghighi and coworkers (2021) demonstrated that targeting specific genes using genome editing tools could bring about cell cycle arrest in gastric cancer cells [215].

Meanwhile, they found that CRISPR/Cas9-mediated knockout of nuclear paraspeckle assembly transcript 1 (NEAT1) in AGS cells eventually caused S phase cell cycle arrest in vitro [215]. NEAT1, as a lncRNAs, contributes to adjusting cell cycle progression, apoptosis, cell growth, proliferation, and migration in various cells [216, 217]. Also, ablation of NEAT1 triggered apoptosis of AGS cells, in part by upregulation of FAS level, thereby eliciting caspase cascade activation [215]. Besides, other reports have indicated that knockout of the EGFR mutation vIII (EGFRvIII) may target glioma cells’ pathogenesis [218, 219]. It seems that EGFRvIII ablation abrogates NF-κB activation in glioma cells and may thereby improve the overall survival rate in glioma patients [220]. Given the positive association between the expression of tumor vascular laminin-411 (α4β1γ1) with potentiated tumor growth and with the expression of cancer stem cell (CSC) markers, other studies have focused on targeting its expression to assess its role in glioma models [221, 222]. Elevated levels of laminin-411 also have a tight interrelation with increased recurrence rate and shorter survival of glioma patients [223]. Interestingly, KO of the laminin-411 α4 and β1 chains with CRISPR/Cas9 could reduce tumor growth in glioma cell-bearing mice and considerably improve their survival because of downregulation of the Notch pathway [224]. Concerning the assumed hypothesis indicating that Notch signaling can stimulate glioma aggressiveness, targeting up- or downstream of Notch could be a rational approach to alleviate disease progression in vivo [224].

## CRISPR/Cas9 in CAR-T cell therapies

Chimeric antigen receptors (CARs) have been applied to genetically engineer T effector cells to potentiate adoptive cellular therapy (ACT) and tumoricidal activities [225]. CARs as recombinant synthetic surface receptors can recognize a specific target antigen on the surface of cancer cells, and subsequently bring about the induction of redirected effector cells activation. The basic CAR construct is made up of a single-chain variable fragment (scFv; ectodomain) that serves as an extracellular antigen-recognition domain [226, 227]. Meanwhile, CAR-T cell therapy has resulted in excellent outcomes in the treatment of a variety of hematological malignancies including acute lymphoblastic leukemia (ALL), chronic lymphocytic leukemia (CLL), lymphoma, and multiple myeloma (MM) [228]. Additionally, CAR-T cell research and development has shown great promise in solid tumors including melanoma, NSCLC, breast cancer, and sarcoma [229, 230].

**Fig. 3:**
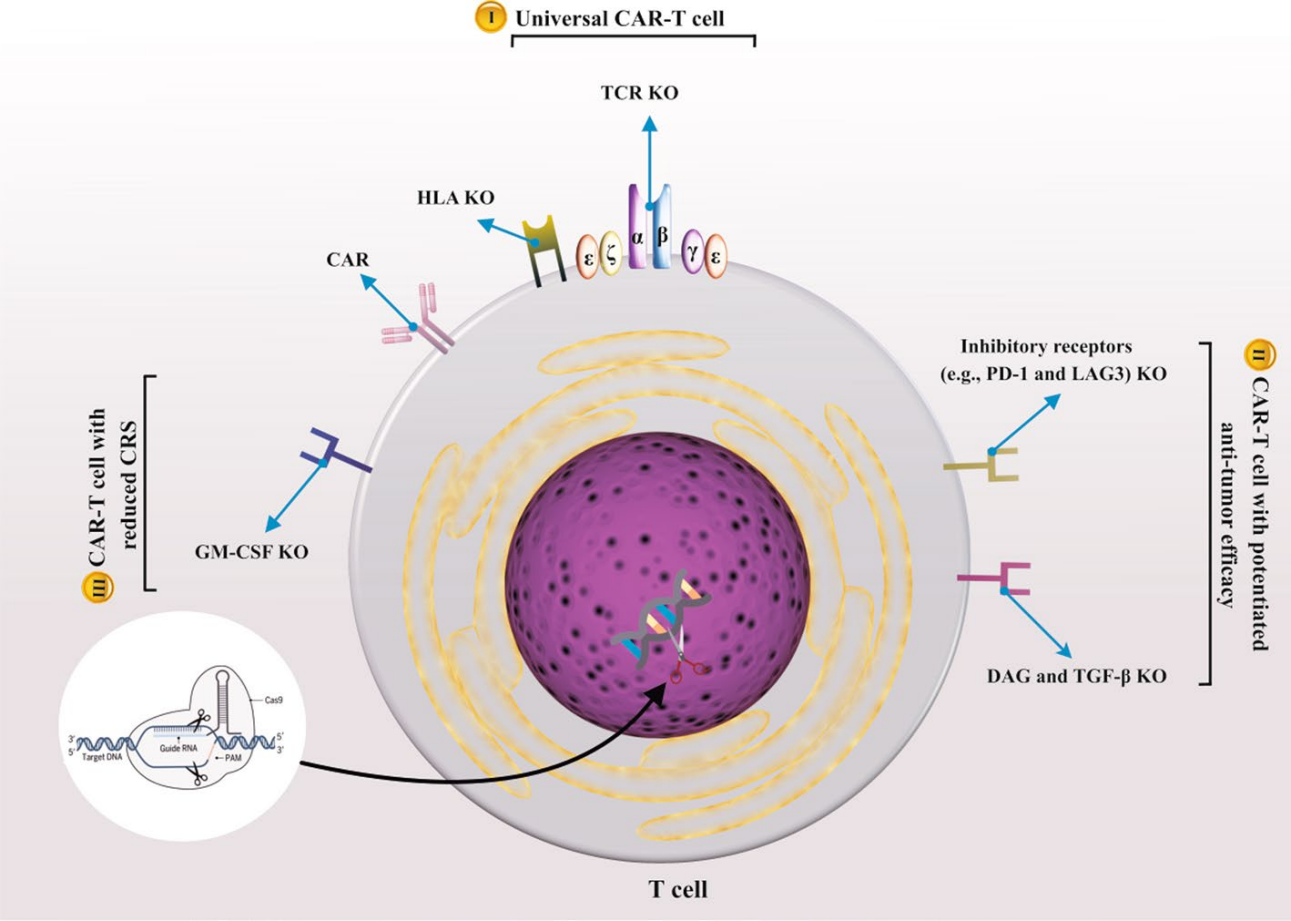
CRISPR/Cas9 application for manufacture of next-generation CAR-T cells. Knockout (KO), T cell receptor (TCR), chimeric antigen receptor (CAR), human leukocyte antigen (HLA), granulocyte–macrophage colony-stimulating factor (GM-CSF), programmed cell death protein 1 (PD1 or PDCD1), lymphocyte activating gene 3 (LAG-3), transforming growth factor-beta receptor (TGF-βR), diacylglycerol (DAG)

Despite this groundbreaking success, obstacles to CAR-T cell therapy include three main challenges: (1) the need for case-by-case autologous CAR-T cell generation, (2) cancer cell resistance to CAR-T cell therapy, and (3) occurrence of unwanted toxicities and, more importantly, cytokine release syndrome (CRS) [231]. The need to create autologous CAR-T cells on a case-by-case basis prevents its large-scale clinical application due to the expensive and lengthy manufacturing process [232–234]. Induced CAR-T cells could express immune checkpoint molecules such as PD1 and lymphocyte activation gene 3 (LAG3) or CD223, thus deterring CAR-T anticancer function upon interaction with corresponding ligands expressed by cancer cells [235, 236]. Activating a significant number of CAR-T cells concurrently and secretion of higher levels of GM-CSF, IL-6, and IL-1 may bring about CRS [237]. Generating off-the-shelf, allogeneic CAR-T cells with robust resistance to immunosuppressive TME accompanied by lower toxicity is urgently required (Fig. 3) (Table 4).

**Table 4:** CRISPR/Cas9 applications in CAR-T cell therapy

Cancer	Target locus	Cell/animal	Delivery method	CAR	Ref.
Ewing sarcoma	EZH2	VH-64, RM-82, and WE-68 cell lines Mice	Lentivirus	GD2	[317]
Lymphoma	LAG-3	K562 and Raji cell lines Mice	Plasmid	CD19	[244]
Leukemia	TRAC	MOLT-3, MOLT-4, HSB-2, and CCRF-CEM cell lines	Electroporation	CD7	[318]
Glioma	DGK	U87MGvIII cell line Mice	Lentivirus	EGFRvIII	[319]
Leukemia	GM-CSF	Nalm-6 cell line	Lentivirus	CD19	[250]
Glioma	PD1	U87MGvIII cell line Mice	Electroporation	CD133	[242]
Solid tumors	TGFβRII	HepG2 cell line Mice	Electroporation	Mesothelin	[245]
Lymphoma	TRAC PD-1	NALM6 cells	Electroporation	CD22	[320]
Leukemia Prostate cancer	TRAC, TRBC, FAS, CTLA-4 B2M, PD1	Mice	Electroporation	PSCA, CD19	[321]
Glioma	PD1	U-251MG and Ev-DKMG cell lines	Plasmid	EGFRvIII	[322]
Leukemia	TRAC	Mice	Electroporation	CD19	[323]
Solid tumors	A2AR	E0771, 24JK, MC38, VCAR-3, MCF7, and MDA-MB-435 cell lines	Electroporation	Lewis HER2	[324]

Lymphocyte-activation gene 3 (LAG-3), granulocyte–macrophage colony-stimulating factor (GM-CSF), epidermal growth factor receptor variant III (EGFR vIII), transforming growth factor-beta receptor II (TGFβRII), A2A adenosine receptor (A2AR), prostate stem cell antigen (PSCA), enhancer of zeste homolog 2 (EZH2), diacylglycerol kinase (DGK), cytotoxic T-lymphocyte-associated protein 4 (CTLA-4), T cell receptor alpha/B constant (TRAC/TRBC), beta-2-microglobulin (B2M), human epidermal growth factor receptor 2 (HER2), programmed cell death protein 1 (PDCD1 or PD1)

### Off-the-shelf or universal CAR-T cells

Recent reports have shown that genetic depletion of T cell receptor (TCR) alpha constant (TRAC or TCR) and β-2 microglobulin (B2M), a component of MHC class I molecules (MHC-1 or HLA-1), by CRISPR/Cas9 may efficiently enable generation of universal CAR-T cells [238]. KO of β2M or TARC impairs allogeneic cell recognition by the host immune system and ultimately permits the manufacture of CAR-T cells from allogeneic T cells isolated from healthy donors [239]. For instance, TCR-deficient allogeneic T cells expressing anti-CD7 CAR could induce remarkable cytotoxicity against CD7-expressing leukemia and lymphoma cells in vivo without graft versus host disease (GvHD) occurrence [240]. Also, anti-CD19 CAR-T cells with depleted TCR and B2M did not provoke GVHD but retained antitumor responses in immunodeficient mice [241]. Thereby, CAR-positive TCR-negative T cells could be a reliable plan to establish next-generation CAR-T cells.

### CAR-T cells with higher efficacy

The expression of immune checkpoints such as PD-1 and LAG3 and immunosuppressive biomolecules such as TGF-B in TME prevents significant and long-term activation of CAR-T cells in vivo. In 2019, Hu et al. showed that CRISPR/Cas9-mediated KO of PD-1 in anti-CD133 CAR-T cells resulted in potentiated proliferation and cytotoxicity in vitro and a murine glioma model [242]. In addition, PD-1-deficient anti-EGFRvIII CAR-T cells could stimulate a more efficient antitumor impact on EGFRvIII-positive glioblastoma cells with no adverse effect on T-cell phenotype or other biological activities [243]. Zhang et al. (2017) also showed that LAG-3 KO CAR-T cells exerted vigorous antigen-specific antitumor effect in a murine xenograft model of refractory B cell malignancy [244].

Given the existence of TGF-β in TME, many efforts have been made to establish TGFβ-receptors (R)-deficient CAR-T cells. Various reports have shown that CRISPR/Cas9-mediated genetic depletion of TGF-βRII causes upregulation of receptor tyrosine kinase-like orphan receptor 1 (ROR1) [245], B-cell maturation antigen (BCMA) [246], mesothelin [245], and PSMA [247], mediating specific CAR-T cell-induced antitumor activity by alleviating TGF-β.

### CAR-T cells with minimized CRS occurrence

Due to its role in CRS development, granulocyte–macrophage colony-stimulating factor (GM-CSF) KO CAR-T cells have been suggested as a putative strategy to minimize CRS occurrence upon CAR-T cell administration [248]. GM-CSF is secreted at high levels by activated CAR-T cells and primarily contributes to activating monocytes and macrophages [249]. Sterner et al. (2019) displayed that GM-CSF KO CD19-specific CAR-T cells secreted lower GM-CSF in vivo, elicited more efficient antitumor activity, and improved OS in mice treated with GM-CSF-deficient CAR-T cells compared with mice treated with conventional CAR-T cells [250]. Preliminary clinical outcomes of one patient with non-Hodgkin’s lymphoma (NHL) and two patients with multiple myeloma (MM) treated with GM-CSF/TCR KO CAR-T cells demonstrated that CRISPR/Cas9-mediated ablation of GM-CSF/TCR had no adverse effect on CAR-T cell proliferation in these patients [251]. CRISPR-edited GM-CSF/TCR KO CAR-T cells exhibited marked persistence following administration and could reexpand following antigen exposure [251]. Noteworthy, all three patients treated with GM-CSF/TCR KO CAR-T cells attained complete response [251].

## The off-target effect of CRISPR/Cas9

Although various CRISPR/Cas system classes have been developed, their wide-ranging application may be obstructed by various issues [252]. The main drawback of CRISPR/Cas9-driven gene editing is the correct prediction of its off-target function [253]. Off-target effects can be defined as accidental cleavage and mutations at untargeted genomic regions displaying a similar but not identical sequence compared with the target site. Indeed, a high incidence of off-target cleavages (≥ 50%) of RNA-guided endonuclease (RGEN)-stimulated mutations at sites other than the anticipated on-target site is the most eminent concern [254]. Another consideration for CRISPR/Cas9-directed gene editing is its editing efficiency [255]. For proficient gene editing treatment, efficient endonuclease accompanied by a dependable delivery system is paramount [256].

Various plans and methods have been designed and developed to improve the on-target effects and decrease possible off-target effects. Meanwhile, much effort has been invested in alleviating the off-target activity of CRISPR/Cas9 by creating multiple CRISPR/Cas systems that offer better fidelity and accuracy [257]. The genomic frameworks of the targeted DNA associated with the secondary structure of sgRNAs and their GC content (40–60% preferably) play a crucial role in defining the cleavage efficiency; the design of fitting sgRNAs with high on-target function using specific tools is urgently required [257]. In addition, the study of the cleavage potential of 218 sgRNAs using the in vitro mismatch cleavage assay or Surveyor assay signified that nucleotides at both PAM-distal and PAM-proximal site of the designed sgRNA are closely associated with the on-target efficiency [254]. For instance, G (but not C) is favored, and as the first base is closely neighboring the PAM, C (but not G) is favored at position 5 (the fifth base proximal to PAM) [258]. Furthermore, the distance between the PAM site and the start codon considerably varies the cleavage efficiency and target specificity. Also, adjusting the Cas9–sgRNA complex concentration by titrating the Cas9 and sgRNA delivery quantities is another approach that has been suggested to reduce off-target activity [258]. Of course, promoting specificity by decreasing the transfected DNA quantity may result in a decrease of on-target cleavage. The equilibrium between on-target cleavage effectiveness and off-target impacts thus has to be considered [258]. In addition, recent reports have delivered proof of concept that combinations of catalytically inactive Cas9 with endonuclease FokI nuclease domain (fCas9) could edit target DNA sequence with > 140-fold higher than wild-type Cas9 [259]. Further, wild-type Cas9 nuclease could be substituted with the D10 mutant nickase version of Cas9 and paired with two sgRNAs that cut only one strand. The paired nicking approach markedly decreases the off-target activity by 50–1500-fold in vitro [260]. During the last decade, researchers have concentrated on merging designer nuclease development [261], designing computational prediction programs and databases [262], and detecting high-throughput sequencing [263] to recognize off-target mutations and minimize off-target activity. Taken together, minimizing the off-target activity in the CRISPR/Cas9 system undeniably provides solid genotype–phenotype relations, thus enabling the realistic construction of gene editing statistics which, in turn, facilitates the clinical application of these CRISPR/Cas9 tools [258].

## CRISPR screening

**Fig. 4:**
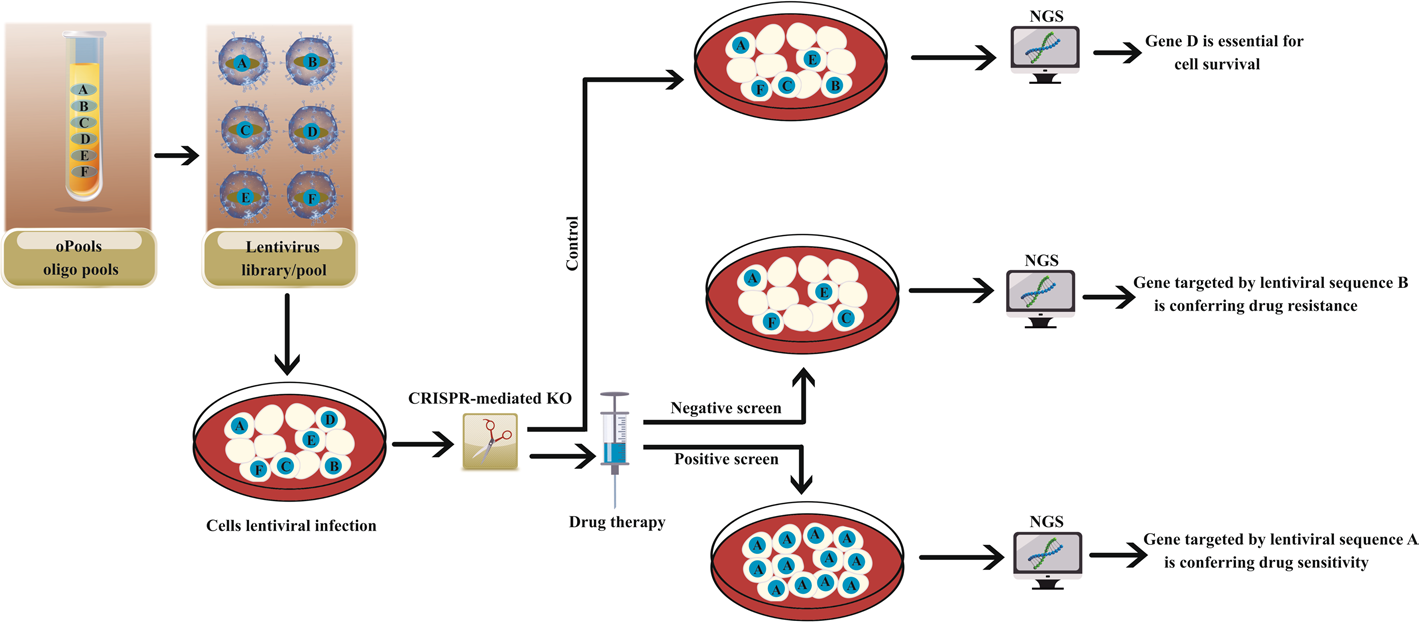
CRISPR screening using pooled DNA oligos. In the most common types of CRISPR screening, a pool of oligos is designed to target a large number of genes. A library of lentiviruses is shaped from the oligos and applied to infect cells. CRISPR genome editing ablates several genes in various cells. Next-generation sequencing (NGS) is applied to define either present or absent genes. Importantly, genes for drug resistance or sensitivity can be detected. Meanwhile, negative screens define genes eliciting resistance, while positive screens define genes eliciting sensitivity

The development of CRISPR screening facilitates high-throughput probing of gene activities in multiple tumor biologies, such as tumor development, metastasis, synthetic lethal interrelation, therapeutic resistance, and response to immunotherapy, which are usually accomplished in vitro or in tumor-cell-bearing animals [264, 265]. CRISPR screening detects essential genes or genetic sequences that largely contribute to stimulating a particular action or phenotype for a cell type [266]. CRISPR/Cas9 exhibits better genetic editing ability, lower off-target effect, and more adaptability. It can be designed and carried out in various formats and affect either coding or noncoding regions in the genome compared with conventional approaches performed using RNAi or cDNA libraries [267]. Although the central idea of CRISPR screening is to knock out every gene (only one gene per cell) that could be significant (Fig. 4), the knockdown screen and activation screen are other types of CRISPR screening with a typical workflow. Firstly, designed sgRNAs are cloned into a lentivirus library and transduced into Cas9-expressing or dCas9-expressing cells at a low multiplicity of infection (MOI) to guarantee that only one copy of sgRNA is integrated per cell [268]. Secondly, CRISPR library-transduced cells undergo biology assay-based screening [268]. If the target gene changes cell fitness in the context of selection pressure, cells containing the sgRNA will be eliminated or potentiated among the population. Lastly, CRISPR screens leverage unique sgRNA sequences and next-generation sequencing (NGS) to detect alterations in sgRNA iteration following phenotypic selection [268]. As such technologies continue to advance, we believe that CRISPR screening will speed up investigations on the functional characterization of genetic materials and the discovery of new therapeutic targets.

## Conclusions and future directions

The CRISPR/Cas9 system allows one to edit a target sequence accurately in model organisms and humans for use in therapeutic analysis. Also, it is theoretically possible to treat infectious and genetic diseases and cancers. CRISPR/Cas9, as a customizable and easily applicable technique, facilitates the enlargement of complete genomic libraries for cancer patients.

Ongoing efforts are planned to maximize its specificity and thus tackle off-target cleavages. The recent progress in the CRISPR/Cas9 methodology reduces undesired mutations. Irrespective of minimizing the off-target action, which is a significant pitfall of gene editing tools, efficient delivery methods that promote their efficacy and constrain immune responses must be developed. Investigators are discovering diverse routes to fine-tune CRISPR delivery to specific cells in the human body. Cas9 ribonuclear proteins (RNPs) are now consistently exploited as a substitute for plasmid vectors for transporting the CRISPR reagent into target cells. This plan potentiates the efficiency, leads to a more transient Cas9 function, and will avert incorporation of vector sequences. Notwithstanding, this strategy does not constrain chromosomal rearrangements. As a final remark, it will be essential to optimize the efficacy, safety, and specificity of CRISPR/Cas9 before its clinical utility.

## Data Availability

Not applicable.

## Abbreviations

CRISPR: Clustered regularly interspaced short palindromic repeat
ZFN: Zinc finger endonuclease
TALEN: Transcription activator-like effector nuclease
CAR: Chimeric antigen receptor
TGFβ: Transforming growth factor beta
sgRNA: Single-stranded guide RNA
crRNA: CRISPR RNA
KO: Knockout
PD1: Programmed cell death protein 1
TCR: T-cell receptor
GM-CSF: Granulocyte–macrophage colony-stimulating factor
LAG3: Lymphocyte activation gene 3
PAM: Protospacer adjacent motif
DSBs: DNA double-strand breaks
